# Implementation of a spike-based perceptron learning rule using TiO_2−*x*_ memristors

**DOI:** 10.3389/fnins.2015.00357

**Published:** 2015-10-02

**Authors:** Hesham Mostafa, Ali Khiat, Alexander Serb, Christian G. Mayr, Giacomo Indiveri, Themis Prodromakis

**Affiliations:** ^1^Institute of Neuroinformatics, University of Zurich and ETH ZurichZurich, Switzerland; ^2^Nanoelectronics and Nanotechnology Research Group, School of Electronics and Computer Science, University of SouthamptonUK

**Keywords:** synaptic plasticity, silicon neurons, memristors, neuromorphic architectures, learning, perceptron

## Abstract

Synaptic plasticity plays a crucial role in allowing neural networks to learn and adapt to various input environments. Neuromorphic systems need to implement plastic synapses to obtain basic “cognitive” capabilities such as learning. One promising and scalable approach for implementing neuromorphic synapses is to use nano-scale memristors as synaptic elements. In this paper we propose a hybrid CMOS-memristor system comprising CMOS neurons interconnected through TiO_2−*x*_ memristors, and spike-based learning circuits that modulate the conductance of the memristive synapse elements according to a spike-based Perceptron plasticity rule. We highlight a number of advantages for using this spike-based plasticity rule as compared to other forms of spike timing dependent plasticity (STDP) rules. We provide experimental proof-of-concept results with two silicon neurons connected through a memristive synapse that show how the CMOS plasticity circuits can induce stable changes in memristor conductances, giving rise to increased synaptic strength after a potentiation episode and to decreased strength after a depression episode.

## 1. Introduction

Biological networks provide a tantalizing proof of the existence of a physically implementable computing architecture that is distributed, fault-tolerant, adaptive, and that outperforms conventional architectures in many important problems such as visual processing and motor control. This has motivated the development of various neuromorphic computing systems whose architectures reflect the general organizational principles of nervous systems in an effort to partially reproduce the immense efficiency advantage that biological computation exhibits in some problems. These neuromorphic systems are organized as populations of excitatory and inhibitory spiking neurons with configurable synaptic connections (FACETS, 2005–2009; Navaridas et al., 2013; Benjamin et al., 2014; Merolla et al., 2014; Ning et al., 2015).

Synapses outnumber neurons by several orders of magnitude in biological neural networks (Binzegger et al., 2004). Reproducing these biological features in neuromorphic electronic circuits presents a scaling problem, as integrating thousands of dedicated synapse circuits per neuron can quickly become infeasible for systems that require a large number of neurons (Schemmel et al., 2007). This scaling problem has traditionally been solved by either treating synapses as simple linear elements and time-multiplexing spikes from many pre-synaptic sources onto the same linear circuit (Benjamin et al., 2014), or by treating them as basic binary elements that can be set either ′*on*′ or ′*off*′ externally, without learning abilities (Merolla et al., 2014).

Real synapses, however, exhibit non-linear phenomena like spike timing dependent plasticity (STDP) that modulate the weight of an individual synapse based on the activity of the pre- and post-synaptic neurons (Bi and Poo, 1998). The modulation of synaptic weights through plasticity has been shown to greatly increase the range of computations that neural networks can perform (Abbott and Regehr, 2004). Capturing the plasticity properties of real synapses in analog neuromorphic hardware requires the use of distinct physical circuits/elements for each synapse. In conventional CMOS, this can lead to restrictions on scalability. Some potential solutions to the scalability issues in pure CMOS technology involve the use of very large integrated structures (e.g., up to a full wafer, Schemmel et al., 2012) or the adoption of deep submicron technologies (Noack et al., 2015). Scalability restrictions however can be greatly relaxed if one resorts to compact nano-scale circuit elements that can reproduce the plasticity properties of real synapses.

One potential candidate for these elements is the “memristor.” Chua (1971) described the memristor as an element which *behaves somewhat like a non-linear resistor with memory*. Since HP first linked resistively switching devices with the concept of a memristor (Strukov et al., 2008), work on memristive devices has mostly focused on digital storage and logic functions (Linn et al., 2012; You et al., 2014), but there are also applications as analog/multi-level storage (Moreno et al., 2010; Shuai et al., 2013) and even memristive encryption (Lin and Wang, 2010; Du et al., 2014). In the neuromorphic community, memristors are seen as ideal devices for synapse implementations, as they combine three key functions in one device. Memristors can implement biologically realistic synaptic weight updates, i.e., learning (Jo et al., 2010), they can carry out long term multi-valued weight storage, and they can also communicate weighted pre-synaptic activity to the postsynaptic side (Saighi et al., 2015), significantly relaxing scalability restrictions (Indiveri et al., 2013).

Typically, plasticity in these memristive synapses is evoked by applying specific waveforms to the two terminals of the memristor, with the waveforms aligned to pre- respectively postsynaptic pulses (Jo et al., 2010). The correlation of the waveforms across the memristor in turn implements STDP-like plasticity (Mayr et al., 2012), with the form of the STDP curve defined by the applied wave shape (Serrano-Gotarredona et al., 2013). Both hardware and software models of plasticity based on the basic STDP mechanism are typically chosen primarily for their simplicity (Mayr and Partzsch, 2010). It has been argued however that more elaborate models of plasticity are required to reproduce the experimental evidence obtained from more complex synaptic plasticity experiments in real neural systems, and to implement algorithms that can learn to store and classify correlated patterns (Senn and Fusi, 2005; Sjöström et al., 2008; Lisman and Spruston, 2010).

In this work we present a neuromorphic implementation of one of these extended plasticity models that implements a spike-based Perceptron learning algorithm (Brader et al., 2007), which makes use of both analog CMOS circuits and TiO_2−*x*_ memristive devices. Compared to the more widely used STDP paradigm, the implementation of this learning algorithm on memristors does not employ the postsynaptic spike timing. Instead, it relies on the correlation of presynaptic spikes with signals derived from the postsynaptic neuron, such as its membrane potential and a measurement of its recent spiking activity. These requirements lead to a novel and quite different approach to the CMOS driver circuits which does not require the generation of temporally long waveforms on the pre- or postsynaptic sides.

In addition to spike timing, plasticity in biological synapses also depends on the firing rate of the post-synaptic neuron (Sjöström et al., 2001), a phenomenon that can not be captured by pair-wise STDP mechanisms (Pfister et al., 2006). The spike-based perceptron learning rule explicitly contains a term that reflects the recent firing rate of the neuron and is thus able to realize the rate-dependence of synaptic weight updates. The rule is also able to realize weight updates that depend on pre-post spike timing even though it does not explicitly depend on the post-synaptic spike times. Instead, it uses the membrane potential of the post-synaptic neuron as an indirect estimator of post-synaptic firing times. The rule is thus able to reasonably match the behavior of biological synapses while having a functional form that can be implemented efficiently on pure CMOS or on hybrid CMOS-memristor neuromorphic systems.

We introduce the spike-based Perceptron learning model in Section 2.1 and the TiO_2−*x*_ memristive devices employed in this implementation in Section 2.2. The adaptation of the learning model to memristors is described in Section 2.3. Considerations for crossbar operation of this paradigm are given in Section 2.4. Section 3.1 shows basic results characterizing operation of the memristors. Characterization of the learning CMOS driver circuits implemented in VLSI are detailed in Section 3.2. Finally, results from implementing the spike-based Perceptron learning with the CMOS driver circuits on the memristors are presented in Section 3.3.

## 2. Materials and methods

### 2.1. The plasticity model

The spike-based Perceptron learning model of long-term plasticity has been introduced in Brader et al. (2007) based on earlier work in Fusi et al. (2000). The model represents a synapse with two stable weights, potentiated and depressed, whereby the transition between the two stable weights is done in an analog or graded manner. The synaptic weight *X*(*t*) is influenced by a combination of pre- and post-synaptic activity, namely the pre-synaptic spike time *t*_*pre*_ and the value of the post-synaptic neuron membrane voltage *V*_*mem*_(*t*) and intra-cellular calcium concentration *C*(*t*). A pre-synaptic spike arriving at *t*_*pre*_ reads the instantaneous post-synaptic values *V*_*mem*_(*t*_*pre*_) and *C*(*t*_*pre*_). The change in *X*(*t*) depends on these instantaneous values in the following way:

(1) $$\begin{array}{l} \text{X}\to \text{X}+a\;if\;\{V_{mem}(t_{pre})>\theta_{V}\;and\\ \;\theta_{up}^{l}<C(t_{pre})<\theta_{up}^{h}\} \end{array}$$

(2) $$\begin{array}{l} \text{X}\to \text{X}-b\;if\;\{V_{mem}(t_{pre})\leq \theta_{V}\;and\\ \;\theta_{down}^{l}<C(t_{pre})<\theta_{down}^{h}\}, \end{array}$$

where *a* and *b* are jump sizes and θ_*V*_ is a voltage threshold. In other words, *X*(*t*) is increased if *V*_*mem*_(*t*) is elevated (above θ_*V*_) when the pre-synaptic spike arrives and decreased when *V*_*mem*_(*t*) is low at time *t*_*pre*_ provided that the calcium variable *C*(*t*) is in the correct range. $\theta_{up}^{l}$, $\theta_{up}^{h}$, $\theta_{down}^{l}$, and $\theta_{down}^{h}$ are thresholds on the calcium variable. The calcium variable *C*(*t*) is an auxiliary variable that is a low-pass filtered version of the post-synaptic spikes (see Brader et al., 2007, for details). The variable *C*(*t*) is incremented by *J*_C_ at each post-synaptic spike time *t*_*i*_, where *J*_C_ reflects the magnitude of spike-triggered calcium influx into the cell. *C*(*t*) decays with a time constant τ_C_:

(3) $$\begin{array}{ll} \frac{d\text{C}(\text{t})}{dt} & =-\frac{1}{\tau_{\text{C}}}\text{C}(\text{t})+J_{\text{C}}\sum_{i}\delta (t-t_{i}) \end{array}$$

The dependence of the weight updates on *C*(*t*) allows the learning rule to enable/disable the weight updates based on the long-term average of post-synaptic activity. *X*(*t*) continuously drifts toward one of two stable values based on whether it is above or below the threshold θ_*X*_:

(4) $$\frac{d\text{X}}{dt}\;=\alpha \;if\text{ X}>\theta_{X}$$

(5) $$\frac{d\text{X}}{dt}\;=-\beta \;if\text{ X}\leq \theta_{X}$$

**Figure 1:**
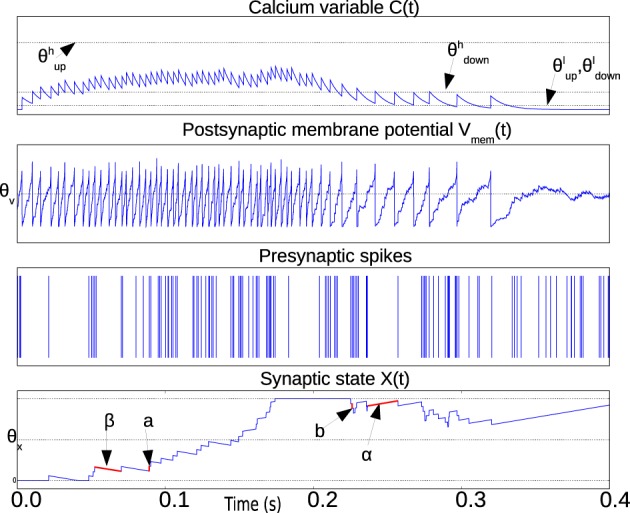
**Illustration waveforms of the spike-based perceptron learning rule showing key parameters from Equations (1–5)**. The Calcium variable plot shows the ranges defined by $\theta_{up}^{l},\theta_{up}^{h},\theta_{down}^{l},\theta_{down}^{h}$ within which synaptic plasticity is active according to Equations (1) and (2). The post-synaptic neuron membrane potential plot shows the threshold θ_*V*_. Incoming synapses can be depressed (potentiated) if *V*_*mem*_(*t*) is below (above) θ_*V*_. The bottom plot showing the synaptic state *X*(*t*) illustrates the jump and drift mechanism. On each pre-synaptic spike, the mutually exclusive conditions in Equations (1) and (2) are evaluated. If the condition in Equations (1) and (2) is fulfilled, the synaptic state jumps up (down) by a step *a* (*b*). The synaptic state is continuously drifting to a high or low state depending on whether it is above or below the threshold θ_*X*_, respectively.

The weight *X*(*t*) is bounded above and below by the two stable states *X*_*high*_ and *X*_*low*_ which are not shown in the equations to simplify the notation. Figure 1 illustrates the relevant waveforms and parameters of the spike-based Perceptron learning rule.

The dynamics of the membrane potential variable, *V*_*mem*_, which is used in Equations (1) and (2), depend on the neuron model used. The original neuron model used with the perceptron-learning rule is the simple constant leak integrate and fire neuron model (Brader et al., 2007). The neuron circuit we have in our neuromorphic chip, however, implements the more realistic adaptive exponential integrate and fire neuron model. This neuron circuit and the underlying model are described in detail in Indiveri et al. (2011) and Ning et al. (2015). The interaction between this adaptive exponential integrate and fire silicon neuron and the spike-based perceptron-learning rule is described in Indiveri et al. (2010).

Although the spike-based plasticity rule described above has been shown to reproduce, on average, the classical STDP phenomenology (Brader et al., 2007), it differs from the vast majority of spike-timing plasticity rules in that it does not explicitly depend on the precise timing of both pre- and post-synaptic neuron spikes. The compatibility with the classical STDP learning rule comes about through the rule's dependence on the post-synaptic neuron's membrane potential: a pre-synaptic spike that occurs when the post-synaptic membrane potential is high will potentiate the synapse and will likely produce a post-synaptic spike shortly after. Thus, the synapse tends to get potentiated in pre-before-post scenarios. The synapse also tends to get depressed in post-before-pre scenarios because the membrane potential is usually low for a few milliseconds after a post-synaptic spike is emitted, and a pre-synaptic spike arriving in this interval will depress the synapse.

The spike-based Perceptron plasticity rule also has access to post-synaptic neuron's rate information through the *C*(*t*) signal. This allows it to reproduce effects beyond classical pair-wise STDP such as increased potentiation at high post-synaptic firing rates and increased depression at low post-synaptic firing rates (Sjöström et al., 2001). These effects arise in more complicated STDP models such as triplet STDP (Pfister et al., 2006; Mayr and Partzsch, 2010). The absence of explicit dependence on the post-synaptic neuron's firing times thus does not diminish the biological plausibility or the computational power of the spike-based Perceptron learning rule.

For the purpose of pure CMOS VLSI implementation (Chicca et al., 2014), this plasticity model is interesting because it can learn a graded response to an input pattern but on long time scales, the weight *X*(*t*) drifts to one of two stable states and is thus easy to store long-term. In the hybrid CMOS-memristor architecture that we propose in this paper, however, the weight drift (Equations 4 and 5) is not implemented. The memristor conductance (weight) only changes on pre-synaptic spikes. Weight drift or the bi-stable synaptic dynamics of the perceptron learning rule can be useful in consolidating the synaptic changes and making the synaptic weight more robust against spurious spikes (Brader et al., 2007). However, this comes at the cost of the sensitivity of the plasticity rule to the temporal spike patterns as multiple spike patterns might lead to the same binary synaptic weights. In the absence of weight drift as in the proposed hybrid CMOS-memristor architecture, the analog synaptic weights are able to maintain a synaptic trace that better reflects the identity of past spiking patterns (Maass and Markram, 2002).

### 2.2. Memristive devices

**Figure 2:**
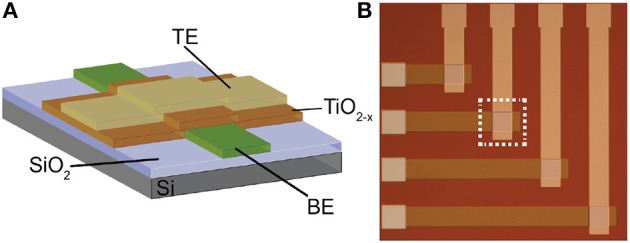
**(A)** Cross-section and **(B)** microphotograph of the memristive device prototype employed in this work.

The memristors that we use as synaptic elements are *TiO*_2−*x*_-based memristors which were fabricated as follows: thermal oxidation was used to grow a 200 nm film of insulating *SiO*_2_ on a 6″ Silicon wafer. Then, bottom electrodes (BEs) were patterned and obtained by conventional optical photolithography, electron beam evaporation and lift-off process. BEs consisted of evaporation of 5 nm adhesive Titanium (*Ti*) and 10 nm Platinum (*Pt*) layers. After that, a similar patterning process was used for the 25 nm *TiO*_2−*x*_ active layer that was deposited in a Leybold Helios Pro XL Sputterer to achieve high quality film. The film was sputtered from a Titanium metal target with 8 sccm flow of *O*_2_, 35 sccm *Ar*, 2*kW* at the cathode, and 15 sccm *O*_2_, 2*kW* at an additional plasma source. Then, again optical photolithography, electron beam evaporation and lift off process were used to pattern and deposit the 10 nm *Pt* top electrodes (TEs). Figure 2 shows a cross-section and microphotograph of *Ti*∕*Pt*∕*TiO*_2−*x*_∕*Pt* memristor prototype (device area: 60 × 60μm).

### 2.3. Circuits for memristive learning

**Figure 3:**
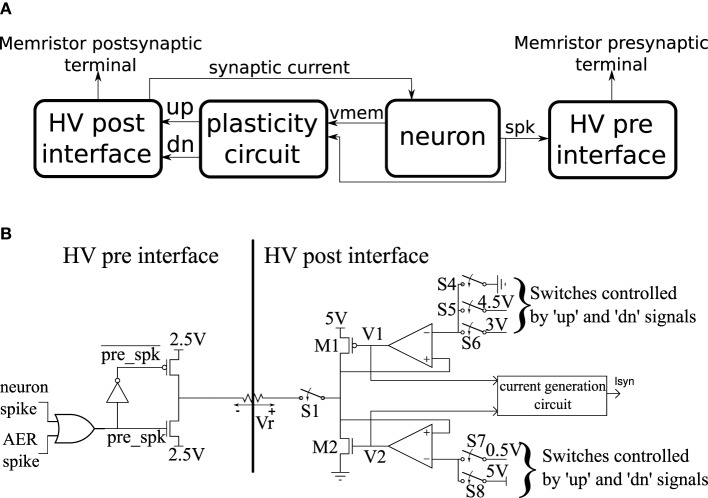
**(A)** High-level schematic of a neuron tile. **(B)** More detailed schematics of the high voltage pre- and post-synaptic interfaces in the neuron tile.

The basic building block of the CMOS circuits is a neuron tile which is shown schematically in Figure 3A. The tile contains an analog subthreshold leaky integrate and fire neuron which is fully described in Qiao et al. (2015). The neuron integrates synaptic current (with an adjustable leak) on a capacitor. When the capacitor voltage crosses the firing threshold, the neuron generates a digital spike and the capacitor voltage is reset to ground. The plasticity circuit monitors the membrane potential, *Vmem*, and the spike output of the neuron and uses them to evaluate the conditions in Equations (1) and (2). The plasticity circuit internally generates the *C*(*t*) signal by low-pass filtering the neuron spikes. The plasticity circuit then generates two digital signals: ′*up*′ and ′*dn*′ that determine whether incoming synapses/memristors should be potentiated, depressed, or left unchanged when a pre-synaptic spike occurs according to Equations (1) and (2). The plasticity circuit is described in more detail in Qiao et al. (2015).

A neuron tile has a pre-synaptic and a post-synaptic memristor terminal. These terminals are monitored and driven by the high voltage post- and pre- interfaces which run at a supply voltage of 5 V. All other circuits operate using a 1.8 V supply. The 5 V operation allows the memristor interface circuits to apply higher voltage pulses to the memristor terminals. The memristor conductance changes if pulses above a certain magnitude (the write threshold) are applied across it. The direction of the change depends on the polarity of the pulse. We designed the interface circuits so that they can interface to memristors having resistance values as low as 1 KOhm and deliver write pulses of either polarity with an amplitude of up to 2 V. The write voltage threshold for the memristor devices we use in this paper is much lower than 2 V. The height of the write pulses are programmable, however, so we can control their amplitudes up to 2 V. The width of the programming pulse is also configurable and can be as wide as 1 ms. The read pulse amplitude (which needs to be below the write threshold) is adjustable in the 0–2 V range and its width is also adjustable. The memristor is inserted between the pre-synaptic terminal of one tile and the post-synaptic terminal of another (or the same) tile. Spikes generated in the neuron circuit of the pre-synaptic tile will then cause a current proportional to the memristor conductance to be injected into the post-synaptic tile neuron. Moreover, based on the output of the plasticity circuit in the post-synaptic tile, a voltage pulse of the appropriate polarity is applied across the memristor terminals to increase/potentiate or decrease/depress its conductance when the pre-synaptic neuron tile generates a spike. In the rest of this section, we describe how this behavior is realized.

The pre- and post-synaptic memristor interfaces are shown in more detail in Figure 3B where they are linked by a memristive element. We retain the ability to disconnect the post-synaptic circuit from the memristor post-synaptic terminal using switch S1. The pre-synaptic memristor terminal is kept floating by default so no current can flow through the memristor and its value remains constant. The post-synaptic terminal is monitoring the current flowing through the memristor and injecting a proportional current into the neuron. By keeping the pre-synaptic terminal floating, no current flows through the memristor, and no current is injected into the post-synaptic neuron. When the pre-synaptic tile neuron spikes, or when the tile receives an AER event from off-chip, the pre-synaptic terminal is strongly clamped at 2.5 V for a short duration that is controlled by an analog bias. By clamping the pre-synaptic terminal to the middle of the supply voltage, we are able to apply pulses of either polarity with an amplitude of up to 2.5 V by setting the appropriate voltage on the post-synaptic terminal. If the post-synaptic terminal is clamped to *V*_*post*_, then on pre-synaptic spike, a pulse of amplitude *V*_*post*_ − 2.5 is applied across the memristor. Assume switch S1 is closed. The post-synaptic terminal can be clamped to one of three possible values: 4.5, 0.5, or 3 V. These clamping voltages can be adjusted through analog biases. The clamping is done by the strong transistors M1 and M2 which are each part of a negative feedback loop that controls their gate potentials so as to maintain their drain potentials at one of the three voltage clamp values. A number of switches which are controlled by the ′*up*′ and ′*dn*′ signals from the plasticity circuit determine which clamping voltage is selected according to Table 1. For example, if switches S5 and S8 are closed and switches S4, S6, and S7 are open, the post-synaptic terminal is clamped by a PFET at 4.5 V. Switches S4–S8 are implemented as single transistors as each switch has to pass a bias voltages that is always either above 2.5 (PFET is used) or below 2.5 (NFET is used). Switch S1 is implemented as a transmission gate.

**Table 1:** **Effect of ′***up***′ and ′***dn***′ signals on the post-synaptic terminal potential which in turn determines the type of plasticity event induced on pre-synaptic spikes**.

**Plasticity signal**	**Vpost**	**Plasticity event**	**Open switches**	**Closed switches**
′*up*′ = 0 and ′*dn*′ = 0	3.0	No change	S4, S5, S7	S6, S8
′*up*′ = 1 and ′*dn*′ = 0	4.5	Potentiate	S4, S6, S7	S5, S8
′*up*′ = 0 and ′*dn*′ = 1	0.5	Depress	S8, S5, S6	S4, S7

*Shown are the open and closed switches in each case. The switches are controlled by the ′up′ and ′dn′ signals*.

At a pre-synaptic spike which causes the pre-synaptic terminal to be clamped to 2.5 V, the memristor experiences a voltage pulse of either 2.0, −2.0, or 0.5 V depending on whether the post-synaptic terminal is at 4.5, 0.5, or 3V respectively. These three cases can either potentiate/increase the memristor conductance, depress/decrease it, or leave it unchanged respectively. It is the plasticity circuit, which through the ′*up*′ and ′*dn*′ signals controls switches S4–S8, which chooses between these three cases (Table 1).

The post-synaptic side indirectly senses the memristor conductance from the gate voltages V1 and V2. When the pre-synaptic side is floating, the two feedback loops push V1 and V2 to 5V and 0V, respectively. The current generation circuit will then generate very little current. At a pre-synaptic event, either V1 or V2 abruptly changes so that the actively clamping transistor has increased effective gate-source voltage so as to be able to source/sink the memristor current while maintaining the drain terminal at the clamp voltage. Larger memristor conductance translates to a larger change in V1 or V2 and based on this change, a proportional current *Isyn* is generated and injected into the post-synaptic neuron. The current generation circuit approximately implements the equation:

(6) Isyn=A∗V2−B∗V1

Where *A* and *B* are constants adjusted through biases. This linear equation is, however, valid in a limited regime of *V*_1_ and *V*_2_. This regime can be adjusted through biases. Note that *Isyn* is proportional to the absolute value of the memristor current, regardless of whether the current is sourced by transistor M1 or sunk by transistor M2. The ′*up*′ and ′*dn*′ signals can not both be high at the same time. For the three possible configurations of the ′*up*′ and ′*dn*′ signals in Table 1, M1 and M2 can not be supplying current at the same time. For the possible configuration of switches shown in Table 1, the feedback loops controlling V1 and V2 ensure that the gate-source voltage of M1 and M2 can not be simultaneously non-zero. This guarantees that the current in either M1 or M2 is the current flowing through the memristor.

This active clamp technique allows maximum voltage headroom for transistors M1 and M2 which allows them to clamp the post-synaptic terminal at voltages near the supply rails. It also enables precise control over the magnitude of the voltage pulses applied across the memristor. In Section 3.2, we present experimental results to illustrate the behavior of the circuit in Figure 3A.

The synaptic weight in the original spike-based Perceptron learning rule has only two stable states due to the weight drift (Equations 4 and 5) which pushes the weight to either a high or a low value. This mechanism is not present in our architecture; the synaptic weight (memristor conductance) is an analog quantity that only changes in response to pre-synaptic spikes and is stable otherwise. Realizing analog synaptic weights that are long-term stable is difficult in pure CMOS as analog weights that are encoded using charge on a capacitor are easily corruptible through leakage paths and capacitive coupling to nearby nodes. Therefore, in pure CMOS, a multi-stability mechanism is required to push the weights to well-defined and stable discrete states. Hybrid CMOS-memristor architectures like ours can realize naturally stable analog weights (memristor conductances) and thus do not require such a mechanism.

**Figure 4:**
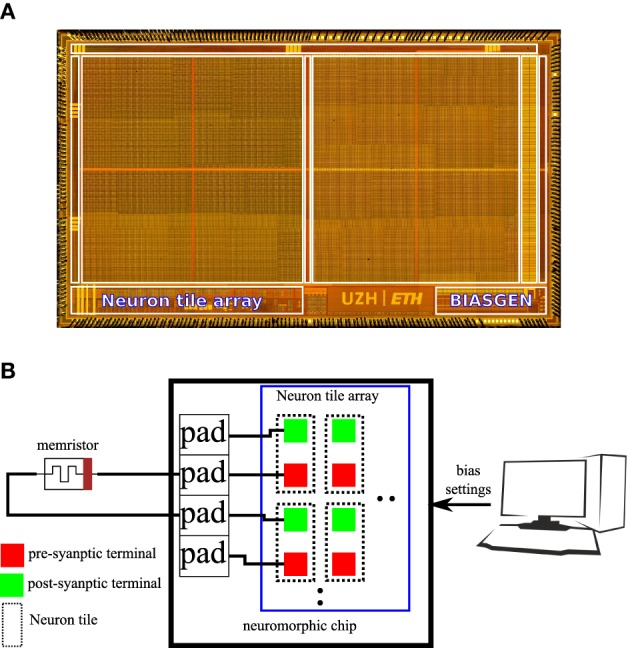
**(A)** Micrograph of the multi-purpose neuromorphic chip die showing the neuron tile array and the bias generator. **(B)** Illustration of the hardware setup used to obtain measurements for this paper. The pre-synaptic terminal of one neuron tile is connected to the post-synaptic terminal of another neuron tile through an off-chip TiO_2−*x*_ memristor. A PC controls the digital settings of the on-chip bias generator.

An 8 * 8 array of the neuron tile shown in Figure 3 was fabricated on a standard 6 M 180 nm CMOS process as part of a larger multi-purpose neuromorphic chip shown in Figure 4A. The chip contains a bias generator based on the design in Delbruck and Lichtsteiner (2006). The bias generator has a low pin-count (5 pins) digital programming interface that can be used to set the values of the analog biases used in the neuron tile array. The other components of the multi-purpose neuromorphic chip are described in detail in Qiao et al. (2015) but they are not relevant for the current paper. Address event representation (AER) interfaces carry spikes to/from the neuron tile array. The pre- and post-synaptic terminals of the 64 neuron tiles were routed to the top-metal level to make it possible to directly deposit a cross-bar array of TiO_2−*x*_ memristors on top that connects a memristor between each pre-synaptic terminal and each post-synaptic terminal. This post-processing step was not carried out. In the chip, the pre- and post-synaptic memristor terminals of two neuron tiles were directly connected to pads. An off-chip memristor was then connected between the pre-synaptic terminal of one of these neuron tiles and the post-synaptic terminal of the other tile as shown in Figure 4B. This setup was used to obtain the measurements presented in the rest of this paper.

### 2.4. Crossbar operation

In a crossbar configuration, *N* neuron tiles are interconnected by an array of *N*^2^ memristors where there is a memristor connecting each pre-synaptic terminal to each post-synaptic terminal. To achieve high synaptic/memristor integration densities, it is important to avoid memristor specific CMOS circuits and only access the memristors through the *N* pre-synaptic terminals and *N* postsynaptic terminals which form the row lines and column lines of the *N* * *N* memristor array. Consider the simple case of *N* = 2 neuron tiles connected using *N*^2^ = 4 memristors. If the post-interfaces in tile 1 and 2 are clamping the post-synaptic terminals to different voltages (which would be the case if one of them is in the ′*up*′ state and the other is in the ′*dn*′ state) then current would flow between the post-synaptic terminals through two memristors connected in series. This would lead to changes in the conductances of these memristors in the absence of pre-synaptic spikes and to synaptic current being mistakenly injected into the neurons. Crossbar operation is thus only possible if plasticity is switched off through the analog biases so that all post-synaptic terminals are clamped at the same potential.

One benefit of using a crossbar array to implement synaptic matrices is that a post-synaptic neuron only needs to know the aggregate input it receives from all synapses rather than the individual contributions. This considerably relaxes the design of the driver circuits as these circuits do not need to isolate the contribution of single devices. Moreover, it has been shown that small selector-less arrays can perform quite well even as analog memory, where good isolation of the contribution of each individual element is required, provided certain assumptions about the switching characteristics of the memristors (e.g., concerning maximum and minimum resistive states) hold (Serb et al., 2015). Thus, even though the current implementation does not take the additional complications of crossbar configurations into account, there is evidence that extension of our work to first small, selector-less arrays and then potentially to larger selector-based arrays is possible.

## 3. Results

### 3.1. Initial memristor programming results

Before the memristors could be used as artificial synapses they were electrically prepared for operation. The preparation procedure consisted of an electroforming step, a stabilization period and a characterization stage. During all stages the devices are biased by a series of square-wave pulses of fixed duration (100 μ s) and variable amplitude.

Initially, the measured resistive state (RS) of all our devices was above the 10 MΩ mark. During electroforming devices were subjected to voltage pulse ramps beginning at 1 V and increasing in steps of 0.5 V until the RS dropped to below 500 kΩ or the maximum limit of 8 V was reached. Typically, electroforming was achieved after applying a 6 V pulse. During electroforming, voltage was applied to our devices through a 100 kΩ series resistor as a measure to protect them against unduly high power dissipation and consequent damage.

In the stabilization period devices are subjected to pulse trains whose amplitudes and polarities are determined by trial and error. During this phase the devices are forced to oscillate between more resistive and more conductive states. This is achieved through use of a bipolar stimulation protocol, that is pulses of opposite polarities drive the RS in opposite directions. During the stabilization period no stable limits for the operational RS ceiling and floor can be reliably determined, nor can appropriate voltages be found at which the memristor will reliably switch between floor and ceiling.

**Figure 5:**
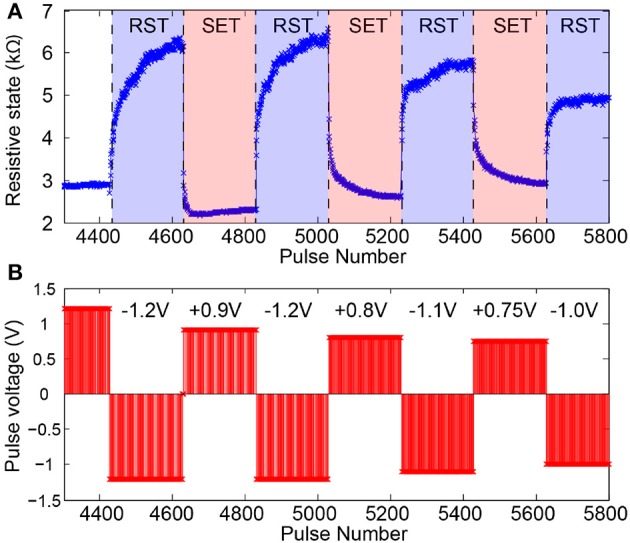
**Memristor switching characteristics during SET-type and RESET-type (RST) pulsing. (A)** Evolution of device under test (DUT) resistive state in reaction to pulsed input stimulation. **(B)** Corresponding pulsing sequence. Pulse width fixed at 100μs.

The characterization stage follows seamlessly from the stabilization period as the device settles to an operational RS range. In this phase voltage pulse amplitudes are trimmed until a set of amplitudes for normal operation biasing is selected. Figure 5 shows a typical characterization stage series of read-outs obtained from a well-behaved device. Notably different voltage values are tested for both SET-type (toward lower RS) and RESET-type pulse polarities within a relatively narrow range (≈ 200 mV). Typically devices can operate comfortably within such narrow ranges although their operational range and the number of pulses it takes to transition between floor and ceiling (and vice versa) will be affected by the exact choice of pulse voltage. See Figure 5 for an example.

Once pulsing voltages have been determined, the memristor may be connected to the appropriately configured neuromorphic circuitry, ready for bipolar-mode operation.

### 3.2. Characterization of CMOS plasticity circuits

**Figure 6:**
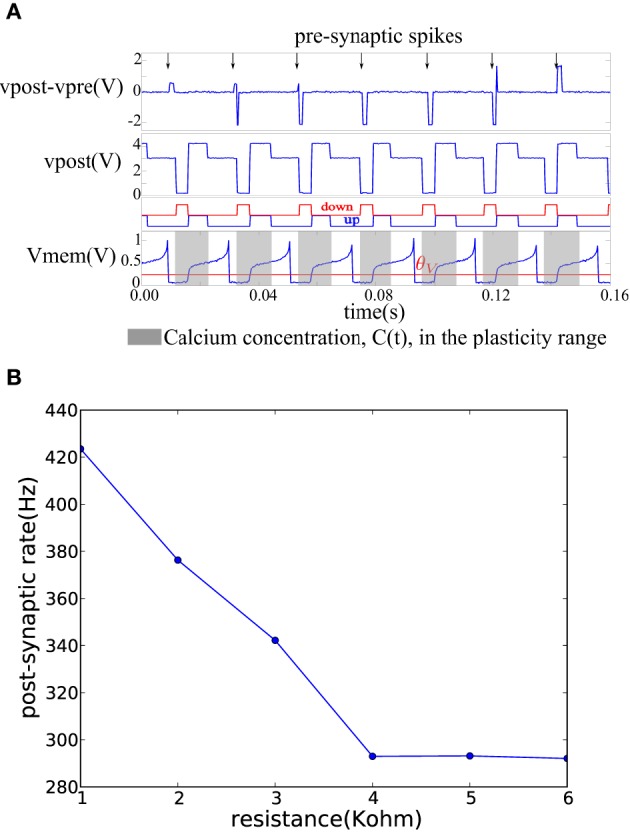
**(A)** Membrane potential and the ′*up*′ and ′*dn*′ signals of a post-synaptic tile receiving spikes at a constant rate from a pre-synaptic tile. The plasticity circuit controls the post-synaptic terminal potential so as to generate an appropriate programming/read pulse across the *vpost* − *vpre* terminals on each pre-synaptic spike. **(B)** Decrease of post-synaptic neuron firing frequency as the conductance of the afferent synaptic element decreases while the pre-synaptic neuron firing rate is kept constant.

On each pre-synaptic spike, the pre- and post-synaptic interface circuits in the neuron tile shown in Figure 3 apply a voltage pulse to update the memristor value according to the spike-based perceptron learning rule. This behavior is illustrated in Figure 6A where a fixed resistor was inserted between the pre-synaptic terminal of one tile and the post-synaptic terminal of another (as in Figure 4B but using a resistor instead of a memristor). Constant current is injected into the neurons to maintain a constant firing rate. The calcium signal, *C*(*t*), jumps up after each spike and enters the plasticity range, then it decays back out of the plasticity range. The bottom two plots in Figure 6A show the ′*up*′ and ′*dn*′ signals and the neuron membrane potential in the post-synaptic tile. The ′*up*′ and ′*dn*′ signals are generated by the plasticity circuit in the post-synaptic tile (see Figure 3A). This plasticity circuit calculates the calcium variable, *C*(*t*), from the post-synaptic neuron spikes according to Equation (3). It evaluates the conditions in Equations (1) and (2) to decide whether to potentiate, depress, or leave unchanged incoming synapses when the pre-synaptic neuron spikes. This decision is communicated to the post-synaptic interface circuit which clamps the post-synaptic terminal voltage *vpost* at 4.1*V* (when the ′*up*′ signal is high), 0.1 V (when the ′*dn*′ signal is high), or 3 V (when both the ′*dn*′ and ′*up*′ are low) as shown in Figure 6A. The pre-synaptic terminal is floating by default and is clamped at 2.5 V for a short duration on each pre-synaptic spike. For each pre-synaptic spike, this causes *vpost* − *vpre* to be approximately 2 V when the ′*up*′ signal is high which would increase the memristor conductance (potentiation), –2 V when the ′*dn*′ signal is high which would decrease the memristor conductance (depression), and 0.5 V otherwise as shown in Figure 6A which would leave the memristor conductance unchanged and simply read out its value. In Figure 6A, at the first pre-synaptic spike, *C*(*t*) is outside the plasticity range and a small read pulse is applied. The subsequent pre-synaptic spikes arrive first in the depression, then in the potentiation intervals of the post-synaptic tile and large amplitude pulses with the appropriate polarity are applied.

Figure 6B shows how the firing frequency of the post-synaptic neuron varies as a function of the value of the resistor connecting it to the pre-synaptic tile. The pre-synaptic tile generates spikes at a constant frequency. The firing frequency of the post-synaptic neuron steadily decreases with the decreasing conductance of the resistor. The bias conditions were chosen to obtain a linear region in the 1–4 KOhm resistance range beyond which the post-synaptic neuron firing frequency saturates at a lower bound. The neuron is biased to have a spontaneous baseline firing rate which is about 290 Hz. The transfer function from the synaptic resistance to the synaptic current injected into the neuron is linear in the 1–4 KOhm region in Figure 6B but beyond that, it is highly non-linear causing a small increase in synaptic resistance to lead to a greatly reduced synaptic current which becomes negligible compared to the constant injection current used to maintain the baseline firing rate. The neuron then saturates at the baseline firing rate.

### 3.3. Memristive plasticity experiments

**Figure 7:**
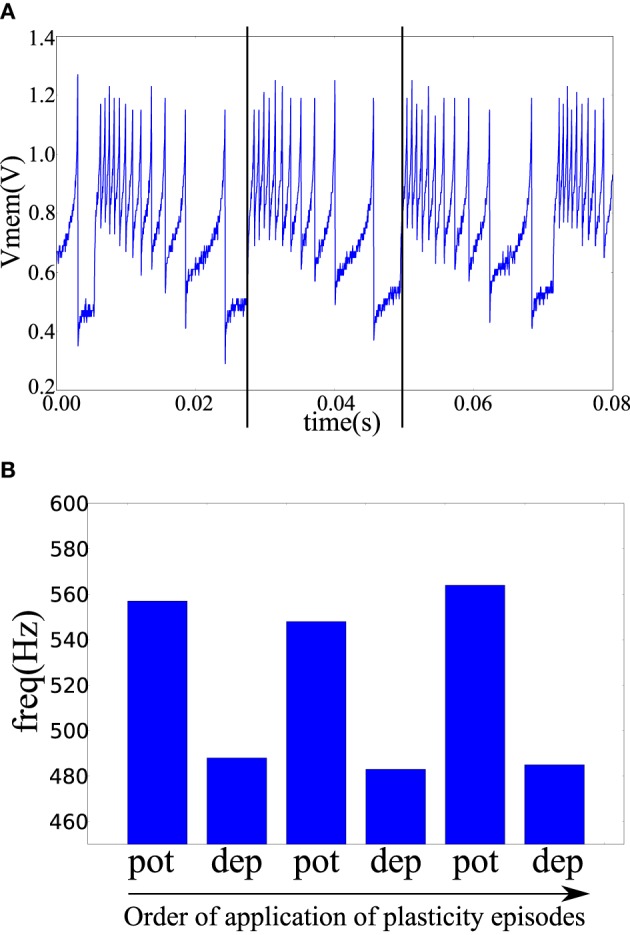
**(A)** The post-synaptic membrane potential in response to a pre-synaptic pulse train of 47 Hz. Vertical lines indicate the times of two pre-synaptic spikes. **(B)** Frequency of the post-synaptic neuron after a number of plasticity episodes. Plasticity episodes were applied in succession by adjusting the analog biases to put the post-synaptic neuron tile in the potentiation and depression regimes.

In the fabricated chip, the pre- and post-synaptic terminals of two neuron tiles were available on the chip pads. The pre-synaptic terminal of one tile was connected to the post-synaptic terminal of the other tile through a *TiO*_2−*x*_ memristor as shown in Figure 4B. The pre-synaptic neuron was biased to fire at 47 Hz. The spike-based perceptron learning circuit was then successively cycled between the potentiation and depression regimes (using the analog biases) and the resulting post-synaptic firing rate was observed. The post-synaptic firing rate was taken as an indication of the synaptic weight or the conductance of the memristive element. The system showed correct operation with the postsynaptic firing rate increasing after a potentiation episode and decreasing after a depression episode as shown in Figure 7.

A potentiation episode involves setting the biases of the plasticity circuit in Figure 3A so that its ′*up*′ output signal is constantly high which will cause the memristor post-synaptic terminal to be clamped at approximately 4.1 V as shown in Figure 6A. On each pre-synaptic spike, a pulse of approximately 2 V is thus applied across the memristor terminals which will act to increase its conductance. Similarly, in a depression episode, the ′*dn*′ output signal is constantly high which will cause a pulse of approximately –2 V to be applied across the memristor terminals on each pre-synaptic spike which will act to decrease its conductance. After each plasticity episode, plasticity was disabled and the post-synaptic neuron firing rate measured, then the next plasticity episode is applied.

## 4. Discussion

### 4.1. Memristive device characteristics

Figure 5 shows the typical operation of a “well-behaved” memristor in response to trains of input voltage pulses. A number of key features are noteworthy:

- *Bipolar operation:* Pulses of opposite polarity precipitate resistive state changes in opposite directions. In the case of our devices, a positive voltage applied to the top electrode (bottom electrode grounded) causes potentiation.
- *Bidirectionally gradual switching:* Transitions between resistive state floor and ceiling occur over many pulses, not just one. This allows the device to work as a multi-level weight artificial synapse (as opposed to binary).
- *Bidirectionally saturating switching:* When a device is bombarded by trains of identical voltage pulses it approaches its operational resistive state floor and ceiling in progressively smaller steps. This implies that the middle of the resistive state range is expected to be most often unoccupied *in operando*, as it is traversed quickly in either direction under pulsing. The resistive state will be therefore multi-level in nature, but most of the time distinctly high or low.
- *Biasing parameter variation tolerance:* The device can remain functional under a relatively wide range of bias voltages. We obtain good switching behavior for voltage pulses in the 0.75–1.2 V range. The device can safely operate with voltage pulses of up to 2 V. This bodes well for operation in tandem with practical electronic systems and for resistive switching behavior tuning.

These features allow the memristive devices to exhibit the correct behavior when coupled to the neuromorphic circuits described in Section 2.3, both as binary and as multi-level synapses. Only binary synaptic operation was investigated in the plasticity experiments.

### 4.2. The spike-based perceptron learning rule in CMOS-memristor architectures

The spike-based Perceptron plasticity rule has been implemented in CMOS neuromorphic systems using various types of circuits such as subthreshold circuits (Mitra et al., 2009) and switched capacitor circuits (Noack et al., 2015). In this paper, we have presented a physical implementation of the first hybrid CMOS-memristor architecture that implements a spike-based Perceptron learning plasticity rule. The physical CMOS-memristor system we presented is a standalone system in which the custom CMOS chip connects directly to the memristive devices. The CMOS chip implements the neuron elements together with dedicated per-neuron circuits that can program (potentiate or depress) the memristive synaptic elements as well as sense their conductances/weights to generate proportional Excitatory Post-Synaptic Currents (EPSCs) in the post-synaptic neuron in response to pre-synaptic spikes. We have presented direct measurements that illustrate the behavior of this physical CMOS-memristor system. This is the first standalone neuromorphic system that combines custom neuron circuits with memristor programming and sensing circuits acting on physical memristive devices.

Many highly accurate and biologically grounded, i.e., non-empirical, synaptic plasticity rules make use of several auxiliary variables beyond spike times in the pre- and post-synaptic neurons to control synaptic weight updates (Pfister et al., 2006; Brader et al., 2007; Clopath and Gerstner, 2010; Mayr and Partzsch, 2010; Graupner and Brunel, 2012). These auxiliary variables may include low-pass filtered versions of the membrane potential (Clopath and Gerstner, 2010) or a low-pass filtered version of the neuron's spike train (Brader et al., 2007). Interestingly, the time difference between pre- and post-synaptic spikes does not figure explicitly in these models. This presents a problem for current neuromorphic memristive architectures that mainly depend on this time difference (through the overlap between pre- and post-synaptic spike-triggered waveforms) to induce weight updates. These architectures will not be able to handle weight updates that are triggered on single pre- or post- synaptic spikes.

The architecture we presented triggers weight updates on single pre-synaptic spikes. This has a significant advantage: at the time of a pre-synaptic spike, the neuromorphic synapse can be immediately potentiated or depressed based on the current state of the post-synaptic neuron; the neuromorphic system does not have to wait for a post-synaptic spike to know the outcome of the plasticity event. Implementations of classical pair-wise STDP rules using memristors typically trigger long waveforms on the pre- and post-synaptic sides of the memristor in response to pre- and post-synaptic spikes respectively. When these waveforms overlap, the potential difference across the memristor exceeds a threshold and changes in memristor conductance occur. The duration of these waveforms dictate the STDP window. The overlapping waveforms paradigm is problematic in the high spike rate regime as multiple spikes can occur within the STDP window, thereby corrupting the synaptic weight update. By contrast this problem is completely avoided in the case of the spike-based Perceptron learning rule.

In the original learning rule (Brader et al., 2007) the weights were bistable, i.e., they gradually drifted to one of two stable states. This had the effect of consolidating synaptic changes and making it more difficult for a synaptic pattern to be corrupted by spurious spikes. Our architecture does not implement such continuous (non event-driven) weight drift. This indicates that synaptic rule features that simplify pure CMOS implementations like bistable weights do not necessarily translate to simpler CMOS-memristor implementations.

### 4.3. Outlook

The architecture we describe represents a first step toward hybrid CMOS-memristor implementations of more elaborate plasticity rules that go beyond standard STDP. Further developments will have to address the problem of plastic crossbar operation as well as mechanisms that allow continuous or non event-driven weight updates.

### Conflict of interest statement

The authors declare that the research was conducted in the absence of any commercial or financial relationships that could be construed as a potential conflict of interest.
